# Corrosion Resistance of Fly Ash-Enhanced Cement-Based Materials in High-Chloride Gas Storage Reservoirs

**DOI:** 10.3390/ma19020406

**Published:** 2026-01-20

**Authors:** Hong Fu, Defei Chen, Bao Zhang, Hongjun Wu, Sheng Huang, Weizhi Tuo, Kun Chen, Hexiang Zhou, Yuanwu Dong

**Affiliations:** 1 R&D Center for Ultra Deep Complex Reservior Exploration and Development, CNPC, Korla 841000, China; catherinefu90@sina.com (H.F.);; 2 Engineering Research Center for Ultra-Deep Complex Reservoir Exploration and Development, Xinjiang Uygur Autonomous Region, Korla 841000, China; 3 Xinjiang Key Laboratory of Ultra-Deep Oil and Gas, Korla 841000, China; 4 Petrochina Tarim Oilfield Company, Korla 841000, China; 5 Donghe Oil and Gas Production Management Area, PetroChina Tarim Oilfield Company, Korla 841000, China; 6 National Key Laboratory of Oil and Gas Reservoir Geology and Exploitation, Southwest Petroleum University, Chengdu 610500, China; 7 Petroleum Engineering School, Southwest Petroleum University, Chengdu 610500, China

**Keywords:** underground gas storage, fly ash, high-salinity formation water, Friedel’s salt

## Abstract

**Highlights:**

**What are the main findings?**

- PFS achieved 28.2 MPa compressive strength at 90 °C with no long-term regression.
- Chloride ingress rate in the fly ash system was reduced to 26.6% of the control group.
- Nanoscale pore refinement decreased permeability by nearly one order of magnitude.
- Reactive alumina promotes Friedel’s salt formation, minimizing internal chloride content.

**What are the implications of the main findings?**

- PFS is a superior sealing material for deep, high-salinity underground gas storage.
- Fly ash effectively mitigates salt corrosion risks in high-temperature wellbore environments.
- The study provides a theoretical basis for optimizing durability in subsurface engineering.

**Abstract:**

This study investigates the use of fly ash to mitigate the long-term performance degradation of Portland cement-based sealing materials in high-salinity environments, such as those found in gas storage reservoirs. We systematically evaluated the evolution of material properties under different temperatures and curing periods. Our integrated methodology combining mechanical tests, microstructural analysis, and chloride migration assessment, reveals a multi-faceted mechanism by which fly ash enhances chloride resistance. The key findings demonstrate that reactive Al_2_O_3_ in fly ash promotes the formation of Friedel’s salt, increasing chemical chloride binding and reducing the chloride ingress rate in the Portland cement–Fly ash system (PFS) to only 26.6% of that in the Portland Cement system (PCS). Concurrently, the pozzolanic reaction consumes portlandite (Ca(OH)_2_), forming stable C-A-S-H gel and refining the pore structure by filling interconnected channels. This nanoscale pore refinement decreased permeability by nearly an order of magnitude. After 90 days of curing in 90 °C saline solution, PFS achieved a compressive strength of 28.2 MPa and maintained an exceptionally low internal chloride content of 0.08 wt.%, demonstrating superior long-term durability. This work clarifies the synergistic mechanisms of fly ash modification and temperature effects, providing a theoretical basis for optimizing sealing materials for deep geological reservoirs and experimental support for the application of fly ash in high-temperature, high-salinity engineering environments.

## 1. Introduction

Underground natural gas storage (UGS) facilities are critical infrastructure for strategic reserves, emergency peak-shaving, and national energy security. Given the urgent need for low-carbon, clean energy in China’s development strategy, depleted hydrocarbon reservoir-type UGS has become the predominant construction model. During the conversion of these depleted reservoirs into UGS, cement-based materials are typically employed to seal wellbore sections. This necessitates that the sealing materials maintain long-term integrity in high-salinity formation waters [1,2,3]. However, these brines contain high concentrations of chloride, sulfate, and metal cations, with ionic concentrations increasing with total dissolved solids (TDS) [4,5].

Chloride ions in concrete pore solution exist in two primary forms: free and bound. Bound chlorides interact with cement hydration products through physical adsorption and chemical binding. Physical adsorption predominantly occurs on the surface of calcium silicate hydrate (C-S-H) gel, where chloride ions are attracted to positively charged sites via electrostatic and van der Waals forces [6]. Chemically bound chlorides result from reactions with hydration products of tricalcium aluminate (C_3_A) and tetracalcium aluminoferrite (C_4_AF), forming Kuzel’s salt (Ca_4_Al_2_(OH)_12_Cl(SO_4_)_0.5_⋅5H_2_O) and Friedel’s salt (Ca_4_Al_2_(OH)_12_Cl_2_⋅4H_2_O) [7,8]. Factors such as aluminum content, water-to-cement ratio, and the type and dosage of mineral admixtures significantly influence the chloride-binding capacity of cement [9]. However, exposure to high-chlorinity formation water can alter the microstructure of cement-based sealants, increasing their porosity and permeability while reducing strength. This degradation can create potential pathways for gas leakage, ultimately compromising zonal isolation and wellbore integrity [10,11].

Supplementary cementitious materials (SCMs), such as fly ash, slag, and metakaolin demonstrate considerable potential for enhancing the chloride penetration resistance and chemical stability of cementitious systems. As evidenced by the work of Chen et al. [12], the incorporation of metakaolin, granulated blast furnace slag, or fly ash can promote the formation of Friedel’s salt, thereby improving the chloride-binding capacity of Portland cement matrices. The long-term corrosion behavior of reinforced concrete under accelerated chloride exposure was investigated by Kazi Naimul Hoque et al. [13] The results indicated that partial replacement of cement with 20% fly ash and 50% slag led to higher corrosion current and mass loss. In contrast, a blend of 20% fly ash and 8% silica fume contributed to a denser matrix, reduced permeability, and superior durability performance. To enhance the chloride resistance of cement-based sealants, Thomas [9] and Sanjuán et al. [14] partially replaced Portland cement with pozzolanic materials, including fly ash, slag, silica fume, and metakaolin. These materials improve performance through two key mechanisms: the pozzolanic reaction refines the pore structure, and the supplementary aluminates they provide promote the formation of Friedel’s salt, thereby enhancing the system’s chloride-binding capacity. However, comparative studies on the long-term evolution mechanisms of SCMs under the coupled conditions of high pressure, high temperature, and high salinity in UGS remain limited. Fly ash, a by-product from coal-fired power plants, is rich in silica (SiO_2_) and alumina (Al_2_O_3_). It is now widely used as a sustainable supplementary cementitious material to enhance concrete durability [15]. Although commercial Portland-pozzolan cements (e.g., Type IP) are widely available and governed by established standards for general construction, they are not designed to withstand the high-temperature and high-salinity conditions of deep UGS wells [16]. The incorporation of fly ash enhances the chloride resistance of concrete through three synergistic mechanisms: (1) The pozzolanic reaction between fly ash and portlandite from cement hydration generates additional C-S-H gel. This refines the pore structure, thereby reducing chloride ion diffusion. Studies show that fly ash can lower the chloride diffusion coefficient of cement by 40–60% [17,18]. (2) The spherical morphology and fine particle size (1–100 μm) of fly ash act as a micro-filler. This improves the packing density of the cement matrix, reduces the connectivity of the pore network, and consequently decreases permeability, hindering chloride ingress [19,20]. (3) The high alumina content in fly ash provides reactive Al_2_O_3_, which facilitates the formation of additional Friedel’s salt, thereby increasing the chemical binding capacity for chloride ions [21,22].

Chindaprasirt et al. [23] observed that incorporating fly ash significantly improves the chloride penetration resistance of concrete, with a positive correlation between fly ash fineness and its effectiveness. Furthermore, Liu et al. [24] attributed this enhancement to microstructural densification caused by the fly ash particles and their hydration products. They reported that fly ash addition substantially reduces chloride deposition and significantly lowers the chloride diffusion coefficient, thereby promoting chloride binding within the cement matrix. However, existing research has primarily focused on the performance of fly ash concrete under standard curing conditions. There remains a scarcity of systematic studies on the long-term performance evolution of Portland cement–fly ash systems under the deep, high-temperature conditions typical of underground gas storage facilities. In particular, the synergistic effects of temperature and time on the mechanical properties, microstructure, and chloride ingress mechanisms are not yet well understood, which currently hinders the optimized application of fly ash in sealant materials for such critical infrastructure.

As summarized in Table 1, although existing studies have confirmed the effectiveness of fly ash in enhancing resistance to chloride attack under standard or mild environments, deep underground gas storage reservoirs are characterized by high temperature, high pressure, and high salinity. Consequently, there remains a lack of systematic research on the long-term performance under these coupled conditions.

Therefore, this study systematically investigates the long-term performance of fly ash-modified cement-based sealants in high-salinity formation water, under typical gas storage temperature conditions ranging from 50 °C to 90 °C. Through immersion tests lasting up to 90 days, we evaluated the evolution of key mechanical properties, including compressive, tensile, and elastic moduli. Microstructural changes and chloride resistance were quantitatively characterized by analyzing porosity, permeability, and chloride corrosion area. Furthermore, XRD and SEM-EDS analyses were employed to elucidate the mechanisms of phase evolution and microstructural transformation under the coupled effects of temperature and time.

## 2. Materials and Methods

### 2.1. Material

**Figure 1:**
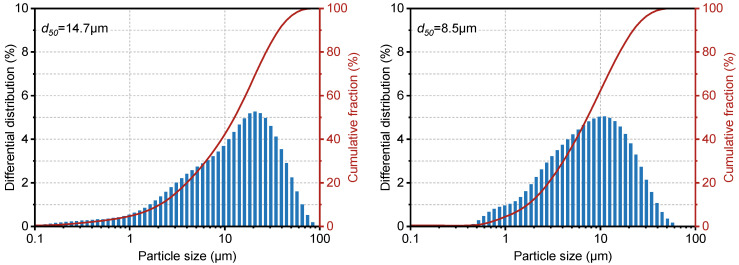
Particle size distribution of Portland cement and Fly ash.

The materials used in this study included Portland cement sourced from Jiasha Special Cement Co., Ltd., in Leshan, China, and fly ash obtained from Lingshou Jinchang Mineral Product Processing Plant in Hebei, in Shijiazhuang, China. A dispersant (SXY-2) was supplied by Chuancheng Chemical Co., Ltd. in Chengdu, Sichuan, China, while a fluid loss controller (G33S) and a retarder (GH-9) were both procured from Weihui Chemical Co., Ltd. in Weihui, China. The chemical compositions of the cement and fly ash were determined by X-ray fluorescence (XRF, PANalytical Axios, Almelo, The Netherlands), as summarized in Table 2. Particle size distribution was measured using a Zeta PALS 190 Plus analyzer, in Shanghai, China (Figure 1). The results indicate that the fly ash had a narrower particle size distribution and was generally finer than the cement, with a median particle size (d_50_) of 8.5 μm. XRF results revealed that, compared to cement, the fly ash contained 5% less calcium oxide (CaO) but 38.7% more SiO_2_ and 45.8% more Al_2_O_3_.

### 2.2. Sample Preparation

The compositional details of the Portland cement–Fly ash system (PFS) and the Portland Cement system (PCS) used in this study are provided in Table 3. The slurries were prepared in accordance with API Recommended Practice 10B. They were then cast into 50 mm × 50 mm × 50 mm cubic and φ 50 mm × 25 mm cylindrical molds and statically cured in a high-pressure curing chamber (OWC-9480C, Applied Technology Co., Ltd. of Shenyang Aerospace University, in Shenyang, China) at 90 °C and 20.7 MPa for 48 h before demolding.

**Figure 2:**
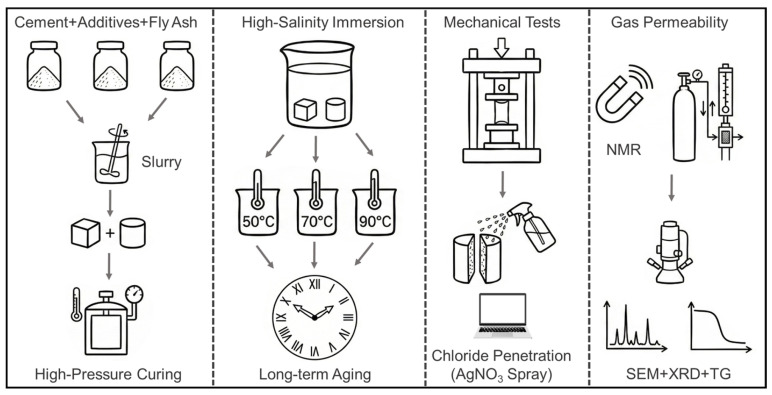
Schematic diagram of the experimental workflow.

The demolded PFS and PCS specimens were immersed in a 3.9 mol/L sodium chloride solution to simulate a high-salinity formation water environment with a total dissolved solids (TDS) content of approximately 228 g/L [29]. To investigate the long-term performance evolution of the cement-based sealants, immersion tests were conducted at 50 °C, 70 °C, and 90 °C for 0, 2, 7, 14, 28, 60, and 90 days. To clearly illustrate the experimental design of this study, a schematic diagram showing the complete workflow, from sample preparation and saline immersion to the subsequent multi-scale characterization is presented in Figure 2.

### 2.3. Characterization of Mechanical Properties

The compressive and tensile strengths of the cured specimens were measured using a fully automatic electro-servo pressure testing machine (YAW-300, Changchun Hao Yuan Testing Machine Co., Ltd., in Beijing, China). The elastic modulus was derived from the uniaxial stress–strain curves. For each test condition, three replicate specimens were prepared. If the deviation between any two results and their average exceeded 15%, the test was repeated with new samples. The loading rates for the compressive and tensile strength tests were set to 6.9 kN/min and 1.0 kN/min, respectively. The elastic modulus was calculated based on the uniaxial compressive strength curve, while the compressive and tensile strengths were determined using Equations (1) and (2), respectively.

(1) $$P_{C}=\frac{F}{S}$$

(2) $$\sigma =\frac{2P_{P}}{\pi dl}$$

**Figure 3:**
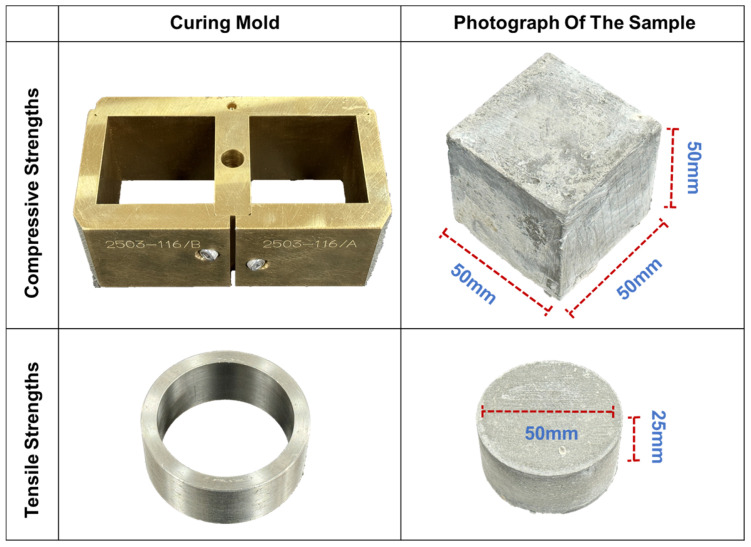
Casting molds and prepared specimens for mechanical testing.

In these equations: *P_C_* is the compressive strength, MPa; *F* is the applied load, kN; *S* is the contact area, mm^2^; *σ* is the tensile strength, MPa; *P_P_* is the peak failure load, kN; d is the specimen diameter, mm; and l is the specimen length, mm [30]. The specific geometry of the casting molds and the physical appearance of the cured specimens used for compressive and tensile strength tests are illustrated in Figure 3.

### 2.4. Characterization of Chloride Ingress Rate

A 0.1 mol/L AgNO_3_ solution was prepared in advance and stored in a brown reagent bottle. After the curing period, the specimens were split vertically to obtain a flat cross-section. While the surface was still moist, it was evenly misted with AgNO_3_ solution to ensure complete coverage. The samples were then exposed to natural light for 10 min to allow the color to develop fully. After the color stabilized, the distribution was recorded via image acquisition. The captured images were analyzed using ImageJ software (Version 2.16) to perform color threshold segmentation, which automatically identified and calculated the area of the white and brown regions. The white areas represent the distribution of free chloride ions in the pore solution [31].

### 2.5. Characterization of Porosity and Permeability

After saturating the samples in distilled water for 24 h, their pore structure characteristics and pore size distribution were analyzed by measuring the transverse relaxation time (T_2_) using a full-diameter core nuclear magnetic resonance (NMR) analysis system (AniMR-150, Shanghai Electronic Technology Co., Shanghai, China). Subsequently, the samples were dried, and their permeability was determined using an ultra-low gas permeability measurement instrument (Low Gas Permeability Measurement 700, Sanchez Technologies, in Viarmes, France) via the nitrogen pulse decay method.

### 2.6. Characterization of Microstructural and Elemental Analysis

The microstructural morphology and elemental distribution of the samples were characterized using a scanning electron microscope (Zeiss Gemini 300, Carl Zeiss AG, Oberkochen, Germany). Before analysis, the sample surfaces were sputter-coated with a thin layer of gold to enhance conductivity. The SEM imaging and EDS analysis were then performed at an accelerating voltage of 15 kV.

### 2.7. Characterization of X-Ray Diffraction

The dried samples were crushed and ground into a fine powder that passed through a 20 μm sieve. The phase composition and crystal structure were then analyzed using an X-ray diffractometer (DX-2700, Haoyuan Instrument, Dandong, China). The measurement was performed in a continuous scanning mode with a 2θ range from 10° to 80°, a step size of 0.02°, and a scanning speed of 5°/min.

### 2.8. Characterization of Thermogravimetric

Thermogravimetric analysis (TGA 2 SF/1100, Mettler Toledo, Greifensee, Switzerland) was performed to determine the relationship between mass and temperature. The measurement was conducted from 30 °C to 800 °C at a constant heating rate of 10 K/min under a nitrogen atmosphere with a gas flow rate of 50 mL/min.

## 3. Results and Discussion

### 3.1. Compressive Strength

**Figure 4:**
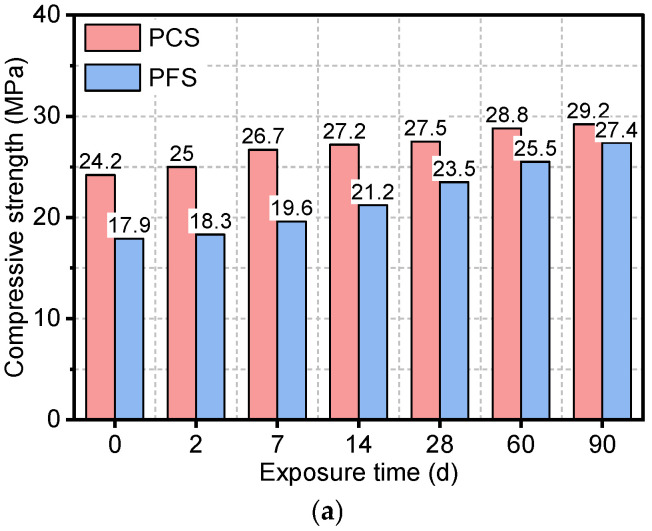

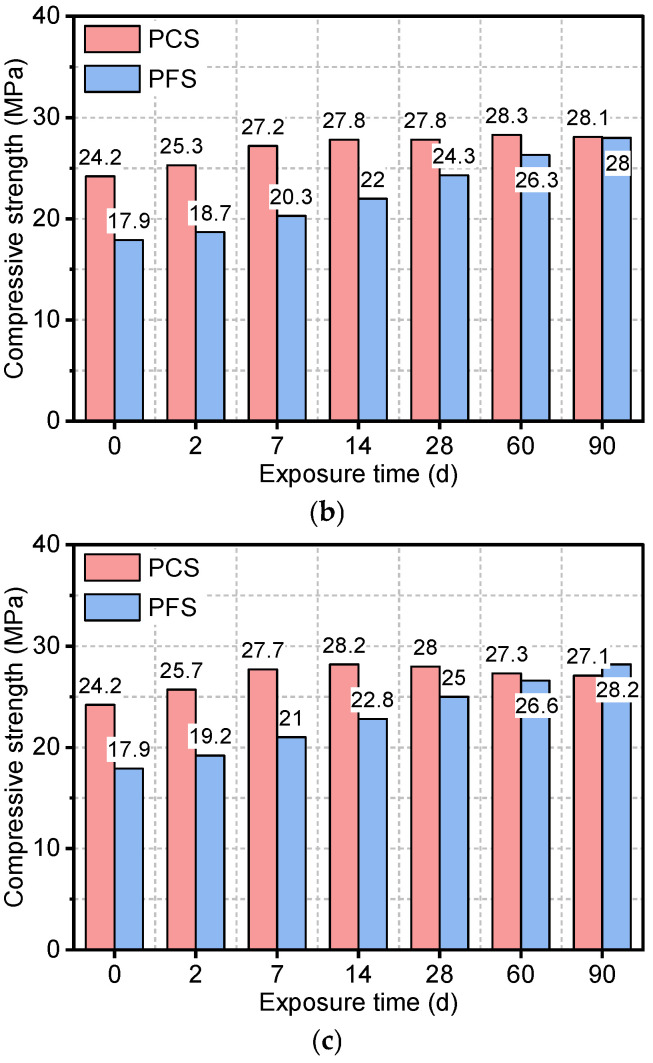
Compressive strength of PFS and PCS after salt corrosion at different temperatures: (**a**) 50 °C, (**b**) 70 °C, (**c**) 90 °C.

Figure 4 illustrates the evolution of compressive strength in the PFS and PCS under different saline immersion temperatures and durations. As shown, for PCS immersed at 50 °C, the compressive strength increased from an initial value of 24.2 MPa to 29.2 MPa after 90 days, representing a 20.7% gain. This strength development was primarily concentrated within the first 14 days, with a marginal increase of only about 2 MPa observed from day 14 to day 90. Elevated temperature had a pronounced negative impact on the long-term strength development of PCS. At 70 °C, although the early-age strength gain slightly surpassed that at 50 °C, a decline commenced after 60 days, resulting in a final strength of 28.1 MPa at 90 days. The strength deterioration was more severe at 90 °C, where the peak strength was attained at 14 days, followed by a continuous decrease. The high temperature not only induces an uneven distribution of hydration products, reducing matrix density, but also accelerates chloride ion ingress. As revealed by the microstructural analysis, the PCS matrix exhibits a loose structure characterized by flaky crystals, which disrupts the integrity of the C-S-H gel. These combined effects lead to the observed mechanical performance regression, posing a significant threat to the long-term durability of the material in high-salinity environments [32].

At 50 °C, the initial compressive strength of PFS was 17.9 MPa, which was 26.0% lower than that of PCS, primarily due to the latent nature of fly ash’s pozzolanic reaction. As the immersion time extended, the strength of PFS increased continuously, reaching 27.4 MPa at 90 days—a gain of 53.1%. At elevated temperatures of 70 °C and 90 °C, the fly ash’s pozzolanic reactivity was significantly enhanced. Here, Ca(OH)_2_ from cement hydration reacted with the reactive SiO_2_ and Al_2_O_3_ in fly ash, generating additional C-S-H and ettringite (AFt) [33]. Consequently, in the 90 °C environment, the PFS strength reached 28.2 MPa at 90 days, surpassing that of PCS under the same conditions. Notably, unlike PCS, PFS showed no strength regression at any temperature, demonstrating excellent long-term stability.

**Figure 5:**
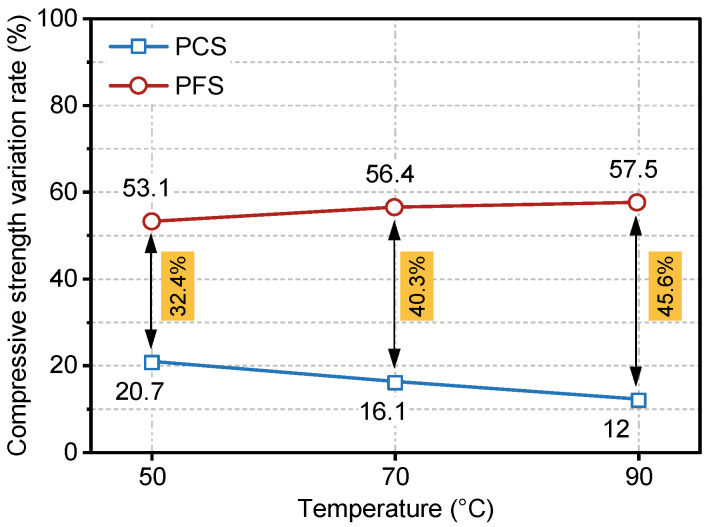
Compressive Strength Variation Rate of PFS and PCS During 0–90 Days of Salt Erosion.

Figure 5 presents a comparative analysis of the evolution in the compressive strength change rate for PFS and PCS during 0–90 days of immersion in saline solution. As shown, the strength change rate of PCS decreased from 20.7% to 12.0% as the immersion temperature increased from 50 °C to 90 °C, indicating a significant deterioration of its load-bearing capacity at elevated temperatures. Conversely, the change rate for PFS increased consistently from 53.1% at 50 °C to 57.5% at 90 °C, demonstrating its ability not only to maintain high strength but also to exhibit a slight enhancement at high temperatures. Furthermore, the performance gap between the two systems, as measured by their change rates, widened from 32.4% to 45.6% with increasing temperature, thereby confirming that PFS’s compressive superiority becomes more pronounced at higher temperatures.

Due to fly ash’s low initial reactivity, it primarily exerts a “dilution effect” during the early hydration stage, resulting in significantly lower compressive strength in PFS than in PCS. As curing progresses, the gradual onset of the pozzolanic reaction produces additional products that effectively fill internal pores. This is corroborated by the SEM observations in Section 3.5, which show that the PFS matrix evolves into a dense network of needle-like and fibrous gels, contrasting with the porous structure of PCS. Furthermore, the porosity and permeability tests confirm a nanoscale refinement of the pore structure, with porosity decreasing to 21.4%. This densification of the microstructure directly underpins the enhanced long-term strength. Recent studies have also highlighted that the synergistic effect of micro-filler action and geopolymeric/pozzolanic reactions significantly contributes to the densification of the matrix and the improvement of mechanical properties [34,35]. Consequently, between 28 and 90 days, the compressive strength of PFS became comparable to, and under 90 °C curing even surpassed, that of PCS [36]. Studies indicate that elevated temperatures promote the formation of Ca(OH)_2_ and accelerate reaction kinetics, while also activating the surfaces of fly ash particles, facilitating their participation in the formation of C-S-H and other hydration products. Consequently, increased curing temperature significantly accelerates the pozzolanic reaction, thereby enhancing the later-age strength development of fly ash-incorporated systems. This mechanism adequately explains the more pronounced strength gain observed in PFS at 70–90 °C [27].

### 3.2. Tensile Strength and Elastic Modulus

**Figure 6:**
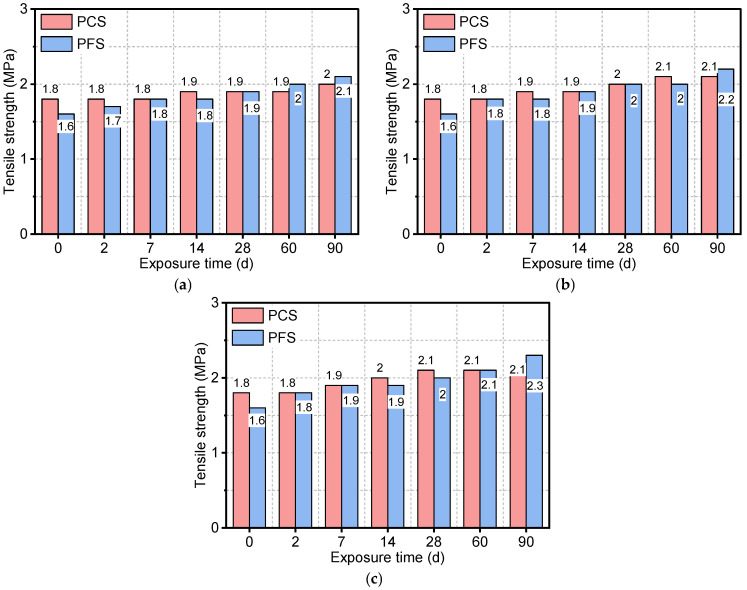
Tensile strength of PFS and PCS after salt corrosion at different temperatures: (**a**) 50 °C, (**b**) 70 °C, (**c**) 90 °C.

Figure 6 illustrates the tensile strength of the PFS and PCS after exposure to saline solutions at different temperatures and durations. As shown, the tensile strength of both systems increased progressively with increasing immersion time, and higher temperatures significantly accelerated this increase. The initial tensile strength of the PCS was 1.8 MPa. After 90 days of immersion at 50 °C, 70 °C, and 90 °C, its strength increased to 2.0 MPa, 2.1 MPa, and 2.1 MPa, respectively. Although the PFS started with lower initial strength, it exhibited a greater strength gain. After 90 days at 90 °C, the tensile strength of PFS increased to 2.3 MPa, representing approximately a 43% increase. This comparison indicates that PFS possesses a greater potential for long-term strength development. The elevated temperature promotes the migration and recrystallization of chloride ions within the pores, and the resulting filling effect further enhances the tensile performance of the PFS [37,38].

**Figure 7:**
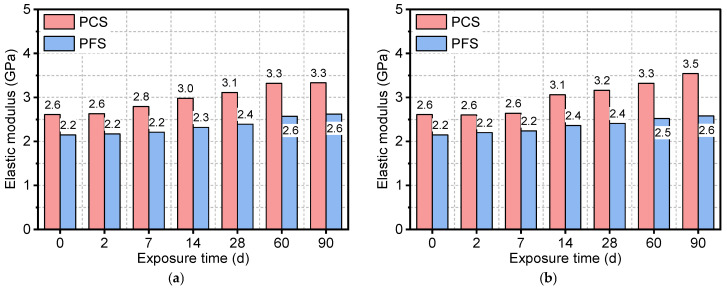

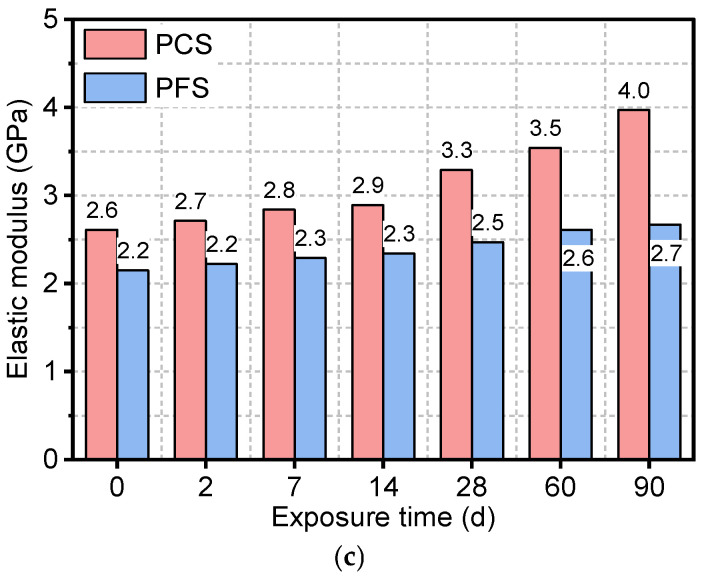
Elastic modulus of PFS and PCS after salt corrosion at different temperatures: (**a**) 50 °C, (**b**) 70 °C, (**c**) 90 °C.

Figure 7 shows the evolution of the elastic modulus for the PFS and PCS under different conditions. The results indicate that while the elastic modulus of both systems increased continuously with immersion time, their rates of change differed significantly. After 90 days in a 90 °C saline solution, the elastic modulus of PCS increased from an initial value of 2.6 GPa to 4.0 GPa, a gain of approximately 50%. In contrast, the development of the elastic modulus in the PFS was more gradual. After 90 days at 50 °C, 70 °C, and 90 °C, its modulus increased from an initial value of 2.2 GPa to 2.6 GPa, 2.6 GPa, and 2.7 GPa, respectively, with a maximum increase of only 25%.

A synthesis of the tensile strength and elastic modulus results reveals that the PCS exhibits a high elastic modulus but lower long-term strength under high temperatures. This is likely due to an imbalance between microstructural degradation from chloride ion erosion and the ongoing hydration process [39]. Specifically, chloride attack can leach calcium from the C-S-H gel, weakening its interlayer bonding. At the same time, the crystallization pressure from salts in the pores generates microcracks that preferentially compromise tensile strength. In contrast, the elastic modulus, which is governed by the solid volume fraction, continues to increase due to new solids formed by ongoing hydration and the pore-filling effects of crystallized salts and phases such as Friedel’s salt [40]. The PFS, however, forms C-A-S-H gel via the pozzolanic reaction of fly ash, offering superior chemical stability. The substitution of aluminum for silicon sites creates Al-O-Si bridging bonds, reinforcing the three-dimensional gel network and enhancing its resistance to dissolution. With its moderate elastic modulus and lower brittleness, the PFS demonstrates greater toughness. This property allows it to dissipate localized stress through slight plastic deformation, thereby avoiding brittle fracture and making it more suitable for engineering environments like gas storage facilities that experience complex stress fluctuations [41].

### 3.3. Analysis of Chloride Ingress Characteristics

**Figure 8:**
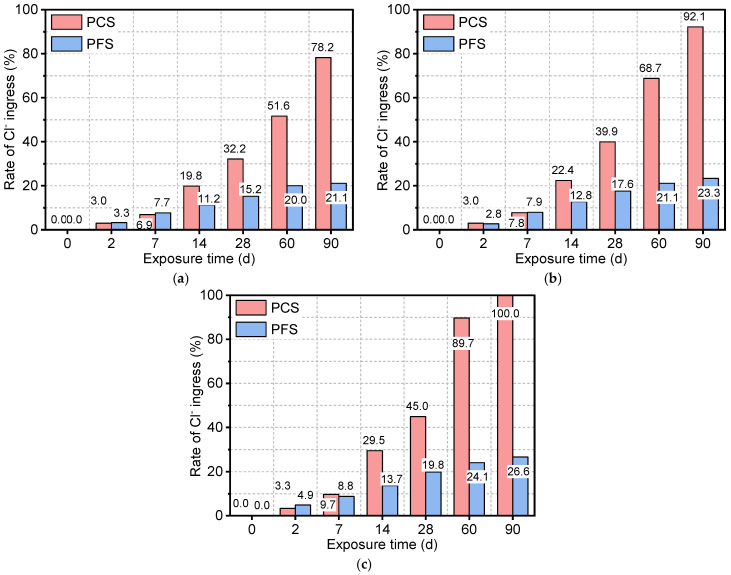
Rate of Cl^-^ ingress of PFS and PCS after salt corrosion at different temperatures: (**a**) 50 °C, (**b**) 70 °C, (**c**) 90 °C.

Figure 8 shows the chloride ion ingress rates for the PFS and PCS under different saline exposure conditions. In a 50 °C solution, the Cl^-^ ingress rate increased rapidly from an initial 3.0% to 78.2%—a 2523% cumulative increase. The ingress was slow in the first 7 days, reaching only 6.9%, but then surged by 368% between days 7 and 28. Higher temperatures markedly accelerated Cl^-^ ingress in PCS; after 90 days, the rates at 70 °C and 90 °C were 17.9% and 27.9% higher, respectively, than the rate at 50 °C. In contrast, PFS demonstrated significantly lower Cl^-^ ingress. After 90 days at 50 °C, its rate was 21.1%, approximately 73.0% lower than that of PCS. Although the two systems had similar ingress rates during the first 14 days, PFS exhibited a much slower rate in the later stages, increasing by only 9.9% from day 28 to 90 compared to 46.0% for PCS. This superior performance is attributed to elevated temperatures, which promote the pozzolanic reaction of fly ash in PFS, refining the pore structure and enhancing chloride binding. Consequently, even after 90 days at 90 °C, the Cl^-^ ingress rate for PFS remained 73.4% lower than for PCS, confirming its excellent long-term resistance to saline corrosion.

The performance gap in chloride resistance between PCS and PFS widens substantially over time. At 50 °C, the difference in Cl^-^ ingress rate was 8.6% at 14 days, which expanded to 57.1% by 90 days. The maximum difference of 73.4% was observed after 90 days at 90 °C. This divergence is attributed to the micro-filler effect of fly ash in PFS, which refines the pore structure and effectively reduces the chloride diffusion coefficient within the cement matrix. According to Fick’s second law, a lower diffusion coefficient directly results in reduced chloride penetration depth and a lower overall ingress rate. Specifically, the mechanism governing chloride ingress in the PFS is twofold: (1) The refinement of pore structure transforms continuous capillary channels into discontinuous pores, drastically reducing the physical pathways for ion diffusion; (2) The abundant reactive Al_2_O_3_ acts as a chemical trap, converting free chloride ions into stable Friedel’s salt, thereby lowering the concentration gradient driving force. This synergistic mechanism fundamentally explains why chloride ingress is effectively arrested in the surface layer of PFS, whereas it penetrates deeply into the porous PCS matrix [42].

### 3.4. Evolution of Porosity and Permeability

**Figure 9:**
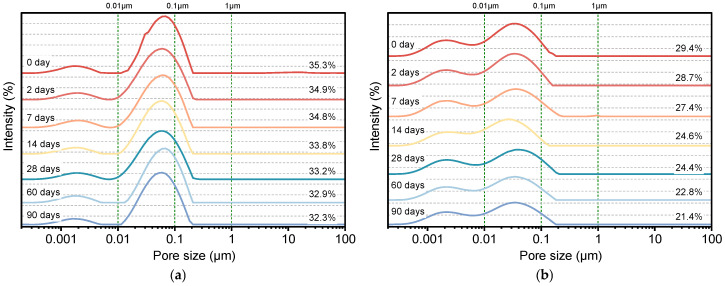
Porosity of PCS and PFS after salt corrosion at different times: (**a**) PCS, (**b**) PFS.

Figure 9 illustrates the porosity evolution of PFS and PCS samples immersed in a 90 °C saline solution over time. The results show that the initial porosity of PCS was 35.3%, which gradually decreased to 32.3% after 90 days—a reduction of 8.7%. In contrast, PFS started with a significantly lower initial porosity of 29.4% and exhibited a more substantial decrease, reaching 21.4% after 90 days. This 27.0% reduction is more than three times greater than that of PCS. These trends demonstrate that incorporating fly ash not only substantially reduces the system’s initial porosity but also effectively refines the pore structure by filling it. Furthermore, the continued hydration of unhydrated cement particles in the saline environment produces additional hydration products that fill pore spaces, thereby further optimizing the material’s microstructure [43].

Based on the classification by Ge et al. [44], pores are categorized by radius into four types: nano-pores (<0.01 μm), micropores (0.01–0.1 μm), mesopores (0.1–1 μm), and macropores (>1 μm). As shown in Table 4, micropores consistently dominate the pore structure of PCS, constituting 75–82% of the total porosity and remaining relatively stable throughout the immersion period. The mesopore content in PCS decreased gradually from 4.6% to 3.2%, while macropores disappeared entirely after 2 days of immersion. In the PFS, the nano-pore content was significantly higher than in PCS. Although it decreased slightly over time, it remained at 8.9% after 90 days. Furthermore, the proportion of micropores in PFS decreased more substantially, from 17.3% to 12.0%—a 30.9% reduction, which is far greater than the change observed in PCS. Notably, no macropores were detected in PFS at any point during immersion.

**Figure 10:**
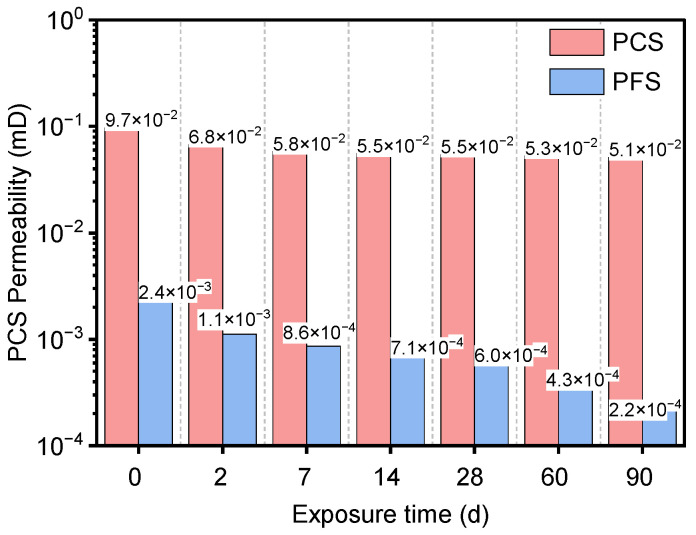
Permeability of PFS and PCS after salt corrosion at different times.

Figure 10 also presents the permeability evolution of PFS and PCS after different immersion durations. The permeability of PCS decreased from an initial value of 9.7 × 10^−2^ mD to 5.1 × 10^−2^ mD, a reduction of 47.2%. A significant 29.8% drop occurred in the first two days, after which the rate of decrease slowed. Permeability theory states that this property is primarily governed by interconnected pores, with macropores and mesopores playing a dominant role [45]. Consequently, the reduction in PCS permeability is closely linked to the rapid disappearance of macropores and a decline in overall pore connectivity. The change in PFS permeability was far more pronounced, decreasing dramatically from 2.4 × 10 ^−3^ mD to 2.2 × 10^−4^ mD, reduction of 90.6%, or nearly an order of magnitude. This decline is much greater than the reduction in total porosity, indicating a fundamental alteration of the pore network connectivity. This sharp decrease in permeability is attributed to two main factors: first, an increased proportion of nano-pores reduces the cross-sectional size of effective flow pathways; second, the pozzolanic reaction of fly ash produces compounds that preferentially fill interconnected channels, thereby effectively blocking the primary permeation paths [46].

In summary, incorporating fly ash significantly enhances the compactness of Portland cement-based materials. By supplying additional Al_2_O_3_, it promotes the reaction of chloride ions to form more Friedel’s salt, thereby reducing overall porosity. This mechanism ensures that the PFS exhibits superior impermeability and long-term structural stability in chloride-rich environments [47].

### 3.5. SEM-EDS

**Figure 11:**
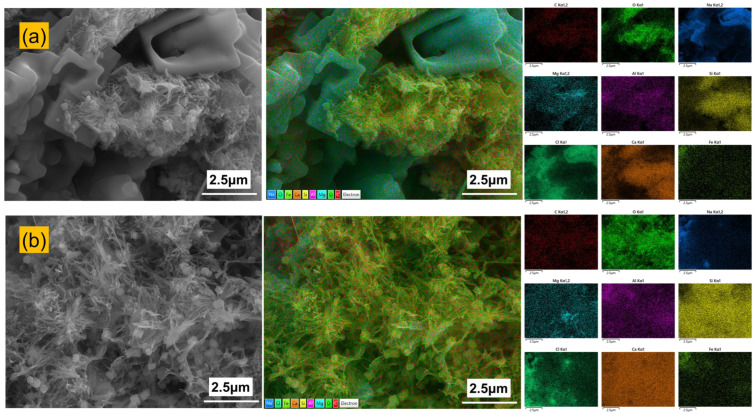
Exterior microstructure and elemental distribution of PCS and PFS: (**a**) PCS; (**b**) PFS.

Figure 11 presents the surface morphology and elemental distribution of PFS and PCS after 90 days of immersion in a 90 °C saline solution. The PCS surface exhibits flaky and platy crystals, along with a small number of needle-like products. In contrast, the PFS surface shows fewer flaky crystals and is primarily composed of a fibrous or needle-like gel. Elemental mapping reveals significant clustering of chlorine on the PCS surface, with its distribution co-located with sodium, indicating the accumulation and crystallization of sodium chloride. Conversely, the distribution of chlorine and sodium on the PFS surface is more uniform, with markedly less enrichment. Combined SEM observation and EDS elemental mapping reveal that the PCS surface contains hydration products and Friedel’s salt, along with a pronounced enrichment of sodium and chlorine. Furthermore, the incorporation of fly ash likely triggered a pozzolanic reaction, producing C-A-S-H and N-A-S-H gels [48].

Table 5 further details the elemental composition of the PCS and PFS surfaces. The PCS surface contains high concentrations of chlorine and sodium at 28.1 wt.% and 13.7 wt.%, respectively, which are significantly higher than typical levels in conventional cement hydration products. This indicates that a large amount of sodium and chlorine is enriched on the surface of the PCS. In contrast, the PFS surface shows 70.9% less chlorine and 29.1% less sodium than the PCS surface. It also exhibits markedly higher calcium and oxygen content, indicating that its surface is predominantly composed of stable hydration products such as Ca(OH)_2_ and C-S-H gel.

**Figure 12:**
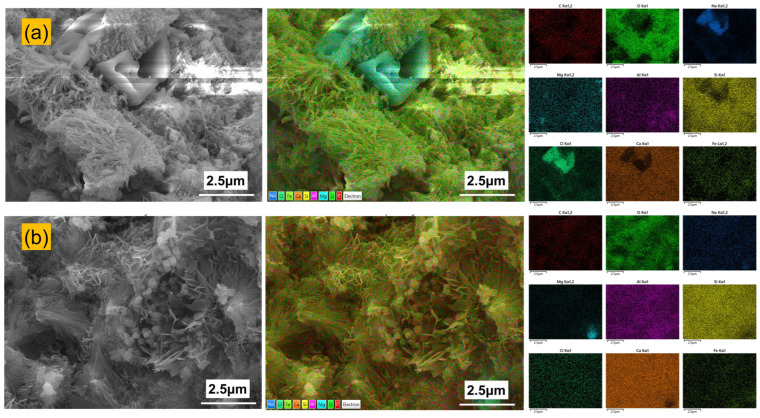
Internal microstructure and elemental distribution of PCS and PFS: (**a**) PCS; (**b**) PFS.

Figure 12 presents the internal microstructure and elemental distribution of PFS and PCS. The internal structure of PCS shows localized needle-like hydration products, whereas PFS is primarily composed of a dense network of needle-like and fibrous hydrates. EDS analysis reveals that the internal elemental distribution of PCS is similar to its surface, with significant concentrations of chlorine and sodium persisting. In contrast, almost no enrichment of chlorine or sodium was detected within the PFS matrix.

Table 6 details the internal elemental composition of PCS and PFS. A comparison with surface data shows that the PCS interior contains 9.8 wt.% chlorine and 5.7 wt.% sodium. In stark contrast, the PFS interior has a chlorine content of only 0.1 wt.%, which is 99% lower than at its surface. This demonstrates that salt ingress is effectively confined to a shallow surface layer. In PFS, the exterior region, which is more saturated, undergoes a more complete pozzolanic reaction, while the interior region retains numerous unreacted fly ash particles. This structural gradient provides a reservoir for long-term strength development, accounting for the increase in PFS compressive strength to 28.2 MPa after 90 days of immersion [49].

### 3.6. Reaction Products

**Figure 13:**
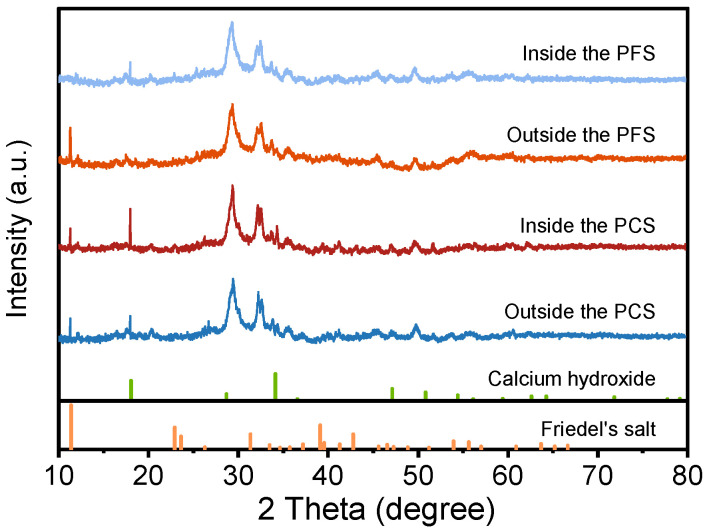
XRD diffraction spectrum of PCS and PFS.

Figure 13 shows the XRD patterns of PCS and PFS after 90 days of curing in a 90 °C saline solution, comparing the crystalline phase composition of their interior and exterior regions. The patterns for both systems show characteristic peaks for Friedel’s salt (2θ = 11.3°) and Ca(OH)_2_ (2θ = 18.0°) [50,51]. Additionally, the diffraction peak near 29.5° is primarily attributed to calcite and residual clinkers such as tricalcium silicate (C_3_S). The signals around 32.7° and 34.4° correspond to unhydrated C_3_A and related intermediate phases, while the peak near 34.1° further confirms the presence of Ca(OH)_2_ [52]. Compared to PCS, the Friedel’s salt peak at 11.3° is significantly more intense in PFS, indicating a greater quantity was formed through the reaction of chloride ions with Al_2_O_3_ and Ca^2+^. Combined with EDS data, this confirms that the reactive Al_2_O_3_ supplied by fly ash promotes Friedel’s salt formation, thereby increasing the chemical binding of chloride ions. Concurrently, the intensity of the Ca(OH)_2_ peak is markedly weaker in PFS, signifying that reactive SiO_2_ continues to consume portlandite via the pozzolanic reaction, producing additional C-S-H and C-A-S-H gels. In summary, incorporating fly ash serves a dual purpose: it enhances the chemical immobilization of chloride ions and facilitates the formation of a denser, more stable gel network.

**Figure 14:**
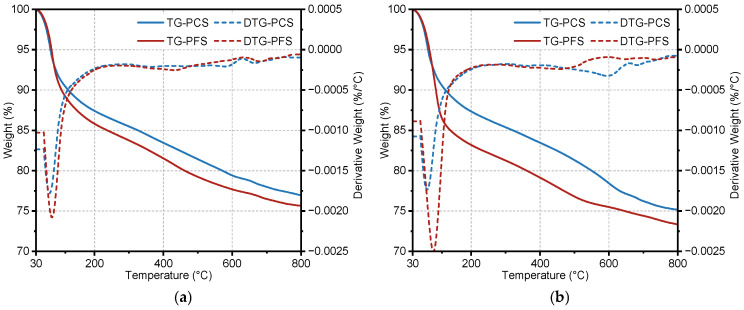
TG/DTG test results of PCS and PFS: (**a**) Exterior; (**b**) Internal.

Thermogravimetric analysis was performed on PCS and PFS samples after curing in a 90 °C saline solution for 90 days (Figure 14). The initial decomposition temperatures for both materials were similar. A significant mass-loss step occurred between 30 °C and 200 °C, during which PFS exhibited greater mass loss than PCS. Within this range, the external mass loss was 12.6% for PCS and 14.2% for PFS, while the internal mass loss was 12.7% and 16.9%, respectively. Over the entire tested temperature range, the total mass loss of PFS remained higher. The total external mass loss was 23.1% for PCS and 24.4% for PFS, and the total internal mass loss was 24.9% and 26.7%, respectively.

The primary products of cement hydration are crystalline or gel-like materials containing water. Upon heating, these hydrates undergo dehydration and decomposition, releasing H_2_O or CO_2_, resulting in measurable mass loss. Each hydrate possesses distinct thermal stability, decomposing within specific temperature ranges with characteristic mass losses. For instance, Friedel’s salt undergoes dehydroxylation between 230 °C and 410 °C, corresponding mainly to the removal of six water molecules from its main structural layers [53]. The Ca(OH)_2_ content, calculated using Equation (3), is presented in Table 7.

(3) $$W_{CH}=\frac{74}{18}\left(L_{1}+\frac{18}{44}L_{2}\right)$$

where *W*_CH_ is the calculated total content of Ca(OH)_2_, %; *L*_1_ is the mass loss within the 440–520 °C temperature range, %; *L*_2_ is the mass loss within the 600–800 °C temperature range, %. The constant 18/44 represents the molar mass ratio of H_2_O to CO_2_, and 74/18 is the molar mass ratio of Ca(OH)_2_ to H_2_O [54].

According to Table 7, the Ca(OH)_2_ content is 10.8% on the exterior and 13.3% on the interior of the PCS sample. This suggests that the external Ca(OH)_2_ was dissolved or transformed by chloride attack, leaving a substantial portion unreacted in the core. In contrast, the Ca(OH)_2_ content in PFS is markedly lower both externally and internally, with a minimal difference of only 0.8% across the sample. This quantitatively demonstrates the extensive consumption of Ca(OH)_2_ by the pozzolanic reaction, which is consistent with the lower intensity of its characteristic XRD peaks in PFS compared to PCS [55]. The mass loss from Friedel’s salt dehydration between 50–280 °C further reveals their differing chloride binding capacities. The greater mass loss on the PFS exterior indicates it formed more Friedel’s salt than PCS. The lower Friedel’s salt content in the PFS interior, compared to its exterior, suggests effective resistance to chloride ingress. Conversely, the similar Friedel’s salt content throughout the PCS sample indicates deep and uniform chloride penetration. This conclusion is strongly supported by EDS analysis, which measured an internal chloride content of 9.8 wt.% in PCS but only 0.1 wt.% in PFS.

### 3.7. Limitations and Future Work

While this study demonstrates the potential of the PFS for sealing UGS wells, several aspects warrant further investigation to facilitate its massive industrial application: (1) The primary hurdle for large-scale deployment is the slower early-strength development of the PFS compared to pure cement, which may prolong the thickening time. Additionally, the inherent variability of fly ash quality requires robust on-site quality control protocols. (2) The current study relied on a pure NaCl immersion environment and a 90-day testing period. However, real formation waters involve complex multi-ion coupling (e.g., Mg^2+^, SO_4_^2−^), and UGS wells require service lives spanning decades.

Based on these findings, we propose the following for future research: (1) Investigate degradation mechanisms under coupled attack from high-salinity brines containing magnesium and sulfate ions. (2) Develop service life prediction models based on accelerated aging tests. (3) Conduct field trials to optimize slurry formulations and validate findings under downhole conditions.

## 4. Conclusions

This study elucidates the mechanism by which fly ash enhances the long-term durability of cement-based sealants in high-temperature saline environments. The key findings are:

(1) Enhanced Mechanical Stability: unlike the PCS which suffered strength degradation, the PFS maintained excellent stability at 90 °C. Its compressive strength increased to 28.2 MPa after 90 days, supported by a dense microstructure that prevented brittle failure.
(2) Superior Chloride Resistance: The chloride ingress rate in PFS was reduced to 26.6% of that in PCS, with negligible internal chloride content (0.1 wt.%). This is attributed to a synergistic mechanism: reactive Al_2_O_3_ promotes Friedel’s salt formation, while the pozzolanic reaction generates C-A-S-H gel to block diffusion channels.
(3) Microstructural Refinement: Fly ash effectively refined the pore structure, reducing porosity to 21.4% and decreasing permeability by nearly an order of magnitude (2.2 × 10^−4^ mD). The transformation of interconnected pores into isolated micropores fundamentally cuts off the migration pathways for corrosive ions.

## Figures and Tables

**Table 1:** Summary of representative studies on the chloride resistance of cement-based materials with SCMs.

Reference	Material System	Environmental Condition	Key Findings
Suryavanshi et al. [25]	PC + Aluminate phases	Standard Lab Condition	Established the fundamental mechanism of Friedel’s salt formation via anion exchange.
Thomas et al. [9]	PC + Fly Ash/Slag	Standard Curing	Fly ash provides reactive alumina to enhance chloride binding and refine pore structure.
Cheewaket et al. [26]	PC + Fly Ash	Marine Environment	Confirmed that fly ash ensures durability over 5 years via pore refinement.
Wang et al. [27]	PC + Low-Ca Fly Ash	Elevated Temperature	Higher temperature accelerates hydration kinetics and modifies Al/Si distribution.
Ali El-Sayed et al. [28]	Class G Cement + Fly Ash + Resin	High-Temp + High-Salinity	Resin reinforcement improves acid/salt resistance in oil well cementing.

**Table 2:** Chemical composition of Portland cement and Fly ash.

Sample	Chemical Composition (%)							
	CaO	SiO_2_	Al_2_O_3_	Fe_2_O_3_	SO_3_	MgO	TiO_2_	Others
Portland cement	65.2	18.8	3.4	5.8	3.2	1.6	0.4	1.6
Fly ash	4.4	45.8	38.7	4.1	2.1	0.6	1.6	2.7

**Table 3:** Sample Formulations.

Sample	Portland Cement (g)	Fly Ash (g)	G33S (g)	GH-9 (g)	SXY-2 (g)	Water (g)
PFS	420	180	12	6	3	300
PCS	600		12	6	3	300

**Table 4:** Pore Size Distribution of PFS and PCS after salt corrosion at different times.

Sample	Exposure Time (d)	Pore Size Distribution			
		<0.01 μm	0.01 μm~0.1 μm	0.1 μm~1 µm	>1 µm
PCS	0	3.4	27.0	4.6	0.4
	2	3.7	27.0	4.2	0
	7	3.7	27.0	4.0	0
	14	3.1	27.1	3.6	0
	28	3.5	25.7	4.0	0
	60	3.2	25.3	4.4	0
	90	2.8	26.3	3.2	0
PFS	0	11.5	17.3	0.6	0
	2	11.2	17.0	0.5	0
	7	11.1	15.1	1.3	0
	14	11.0	13.5	0.1	0
	28	9.8	13.5	1.1	0
	60	9.4	12.7	0.7	0
	90	8.9	12.0	0.6	0

**Table 5:** Exterior elemental composition of PCS and PFS.

Sample	Elemental Composition	Element								
		C	O	Na	Mg	Al	Si	Cl	Ca	Fe
PCS	Weight (wt.%)	6.5	18.2	13.7	0.8	0.8	6.4	28.1	23.6	1.8
	Atomic (at.%)	13.7	28.6	15.0	0.8	0.8	5.8	19.9	14.8	0.8
PFS	Weight (wt.%)	4.9	35.9	4.0	0.8	1.4	9.6	8.2	33.1	2.2
	Atomic (at.%)	9.4	51.6	4	0.7	1.2	7.9	5.3	19.0	0.9

**Table 6:** Elemental composition of the interior of PCS and PFS.

Sample	Elemental Composition	Element								
		C	O	Na	Mg	Al	Si	Cl	Ca	Fe
PCS	Weight (wt.%)	5.0	34.0	5.7	0.5	0.6	10.3	9.8	32.7	1.5
	Atomic (at.%)	9.7	49.2	5.7	0.4	0.5	8.5	6.4	18.9	0.6
PFS	Weight (wt.%)	4.8	43.3	0.3	0.8	3.3	14.6	0.1	31.0	2.0
	Atomic (at.%)	8.6	58.8	0.3	0.8	2.7	11.3	0.1	16.8	0.8

**Table 7:** Mass Loss of PCS and PFS within Specified Temperature Ranges.

Location	Sample	Mass Loss (%)			W_CH_ (%)
		230–410 °C	440–520 °C	600–800 °C	
Exterior	PCS	3.5	1.6	2.5	10.8
	PFS	3.9	1.7	2.1	10.3
Internal	PCS	3.4	1.9	3.3	13.3
	PFS	3.7	1.8	2.2	11.0

## Data Availability

The original contributions presented in this study are included in the article. Further inquiries can be directed to the corresponding author.
